# Magnesium and Human Health: Perspectives and Research Directions

**DOI:** 10.1155/2018/9041694

**Published:** 2018-04-16

**Authors:** Abdullah M. Al Alawi, Sandawana William Majoni, Henrik Falhammar

**Affiliations:** ^1^Division of Medicine, Royal Darwin Hospital, Darwin, NT, Australia; ^2^Department of Medicine, Sultan Qaboos University Hospital, Muscat, Oman; ^3^Menzies School of Health Research, Darwin, NT, Australia; ^4^Northern Territory Medical Program, Flinders University School of Medicine, Darwin, NT, Australia; ^5^Department of Endocrinology, Metabolism and Diabetes, Karolinska University Hospital, Stockholm, Sweden; ^6^Department of Molecular Medicine and Surgery, Karolinska Institutet, Stockholm, Sweden

## Abstract

Magnesium is the fourth most abundant cation in the body. It has several functions in the human body including its role as a cofactor for more than 300 enzymatic reactions. Several studies have shown that hypomagnesemia is a common electrolyte derangement in clinical setting especially in patients admitted to intensive care unit where it has been found to be associated with increase mortality and hospital stay. Hypomagnesemia can be caused by a wide range of inherited and acquired diseases. It can also be a side effect of several medications. Many studies have reported that reduced levels of magnesium are associated with a wide range of chronic diseases. Magnesium can play important therapeutic and preventive role in several conditions such as diabetes, osteoporosis, bronchial asthma, preeclampsia, migraine, and cardiovascular diseases. This review is aimed at comprehensively collating the current available published evidence and clinical correlates of magnesium disorders.

## 1. Introduction

Magnesium (Mg^2+^) has several functions in the human body. It acts as a cofactor for more than 300 enzymes, regulating a number of fundamental functions such as muscle contraction, neuromuscular conduction, glycemic control, myocardial contraction, and blood pressure [1, 2]. Moreover, magnesium also plays a vital role in energy production, active transmembrane transport for other ions, synthesis of nuclear materials, and bone development [2]. Furthermore, magnesium deficiency has been associated with a wide range of diseases. Additionally, many studies have demonstrated beneficial effects of magnesium supplementation. In this review, the magnesium cycle in the human body, magnesium deficiency and its causes, diseases associated with low magnesium, hypermagnesemia, and the role of magnesium in therapy and prevention will be discussed.

### 1.1. Magnesium and Nutrition

According to the United States Food and Nutrition Board, recommended daily allowance for magnesium is 420 mg for adult males and 320 mg for adult females, respectively [3]. Approximately 10% of the daily magnesium requirement is derived from water. Green vegetables, nuts, seeds, and unprocessed cereals are rich sources of magnesium. Also, some magnesium is available in fruits, fish, meat, and milk products [2].

The majority of the population in the Western countries consume less than the recommended amount of magnesium, contributed by the consumption of processed foods, demineralized water, and agricultural practices using soil deficient in magnesium for growing food [3–5].

## 2. Magnesium Absorption and Excretion

Magnesium homeostasis is regulated by the intestines, the bones, and the kidneys [4]. The majority of magnesium is absorbed by a passive paracellular mechanism in the ileum and distal parts of the jejunum, while a smaller amount is actively transported in the large intestine [2, 6]. Around 24–76% of ingested magnesium is absorbed in the gut and the remaining is eliminated in the feces. The proportion of absorbed magnesium from the gut depends on the amount of ingested magnesium [3] and the status of magnesium in the body [2, 4].

**Figure 1:**
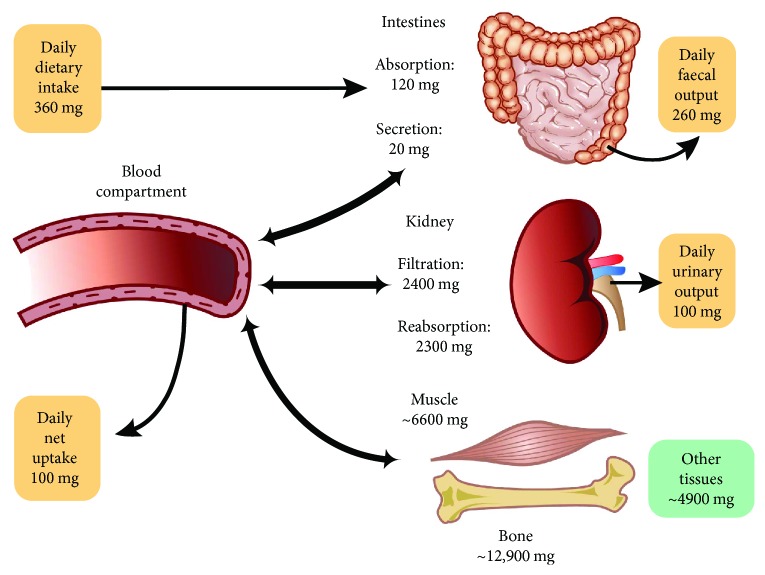
Magnesium balance in the human body.

The magnesium homeostasis is primarily regulated by the kidneys [4]. The glomeruli filter around 2400 mg of magnesium per day. About 95% of excreted magnesium is reabsorbed, mainly by the thick ascending limb of the loop of Henle (65%) and to a lesser extent in the distal tubules (30%) [4, 7]. Only around 100 mg of magnesium is excreted in the urine each day, and the kidneys can regulate the amount excreted, depending on the serum level of magnesium [4].  Figure 1 illustrates the magnesium balance in the human body.

## 3. Role of Hormones in Magnesium Homeostasis

Vitamin D, parathyroid hormone (PTH), and estrogen are hormones that play an important role in magnesium homeostasis (Table 1) [2, 8]. The relationship between PTH and magnesium is complex and similar to calcium; high serum magnesium levels suppress the secretion of PTH via activation of calcium-sensing receptor (CaSR) present on the chief cells of the parathyroid glands. In contrast, low serum magnesium stimulates PTH secretion.

Magnesium has important role in adenylate cyclase activity required for cyclic adenosine monophosphate (cAMP) which is involved in PTH secretion and end organ effects of PTH. Severe hypomagnesemia (<0.4 mmol/L) causes reduced cAMP levels which can result in reduced secretion of PTH and increased peripheral resistance [9, 10]. In addition, molecular studies have suggested that severe hypomagnesemia causes blockage of PTH secretion by disinhibition of G[*α*] subunits and subsequently activation of the CaSR [11]. This paradoxical effect of hypomagnesemia can result in hypocalcemia in patients with severe hypomagnesemia [12]. On the other hand, PTH enhances the reabsorption of magnesium in the distal convoluted tubule and the gut and increases the release of magnesium from the bone [2].

## 4. Magnesium Storage and Circulation

The total body magnesium in the average adult is around 1000 mmol or 24 g, that is, 20 mmol/kg of lean body mass [4]. The bones store about 50–60% of the total magnesium content while muscles and other soft tissues store around 40–50% [3]. Around one-third of the bone magnesium content is available for exchange to maintain the levels of extracellular magnesium [4]. Less than 2% of magnesium in the body is available in serum and red blood cells, accounting for the extracellular magnesium in the body [3].

## 5. Magnesium Transcellular Transportation

Due to the very important role of magnesium in the human body, the levels of cellular magnesium need to be strictly regulated. Several specific transporters controlling the cellular movements of magnesium have been identified [13]. Using the electrochemical gradient of Na^+^ and through cations channels, magnesium enters cells via Mg^2+^/anion cotransport. Eight cation channels have been identified including transient receptor potential melastatin cation channels 6 and 7 (TRPM6, TRPM7), members 1 and 2 (SLC41A1, SLC41A2) channels, ancient conserved domain protein 2 (ACDP2), the mitochondrial RNA splicing 2 protein (Mrs2p), magnesium transporter 1 (MagT1), the human solute carrier family 41, and paracellin-1 [13]. TRPM7 is the most selective channel for magnesium, and it has been identified in the heart, blood vessels, lungs, liver, brain, intestine, and spleen. It is essential for regulating intracellular magnesium level, cell survival, and function [14]. On the other hand, TRPM6 is mainly responsible for regulating the total body magnesium level via the kidney and intestines [15]. Mrs2p, SLC41A1, and SLC41A2 are implicated in magnesium transportation in the mitochondria and hence have a regulatory role related to metabolic, cardiovascular, and neurological functions [16]. Magnesium efflux involves several exchanges including Na^+^/Mg^2+^, Ca^2+^/Mg^2+^, Mn^2+^/Mg^2+^ antiporter, and Cl^−^/Mg^2+^ cotransporter [13]. The most important exchanger is Na^+^/Mg^2+^ exchanger which has been identified in many cells including cardiac and vascular smooth cells. Several factors have been found to effect the function of this exchanger such as vasopressin, angiotensin II, and insulin [13, 17].

## 6. Role of Magnesium in the Human Body

Magnesium is the fourth most abundant cation in the body and the second most abundant intracellular cation after potassium [18]. Magnesium is an essential cofactor for a diverse metabolic reactions involving more than 300 enzymes within the human body [19]. It acts as counter ion for the energy-rich ATP and nucleic acids, regulates transmembrane transport [20], and has various roles in function and structure of proteins, nucleic acid, and mitochondria [2]. Magnesium is an important mineral for bone mineralization, muscular relaxation, and several other cellular functions [18] (Table 2).

## 7. Assessment of Magnesium Status

In clinical practice, serum magnesium concentration is the most commonly used test to assess the magnesium status, and the normal reference range is usually 0.7–1 mmol/L (equivalent to 1.5–2 mEq/L or 1.7–2.4 mg/dL) [21]. However, the normal value varies from laboratory to laboratory, and different studies have used slightly different ranges. This may partially explain the differences in the prevalence of magnesium imbalances reported in different groups of patients with similar characteristics [4].

Normal serum magnesium does not necessarily mean adequate content of total body magnesium because only less than 0.3% of total body magnesium is found in serum [4]. Serum magnesium is in most places not part of routine blood tests, and it should be assessed in the relevant clinical conditions such as arrhythmia, hypokalemia, hypocalcemia, diarrhea, and chronic alcoholism that tend to be associated with magnesium derangement [20]. Assessment is also recommended if the patient is critically ill or when being administered certain medications known to cause hypomagnesemia.  Table 3 lists other more accurate but lesser used measures of assessing magnesium status [2, 22].

## 8. Hypomagnesemia

Several studies have shown that hypomagnesemia is a common electrolyte derangement in clinical setting especially in patients admitted to intensive care unit (ICU) where it was found to be associated with increased mortality and hospital stay [23–25] (Table 4).

### 8.1. Symptoms of Hypomagnesemia

**Figure 2:**
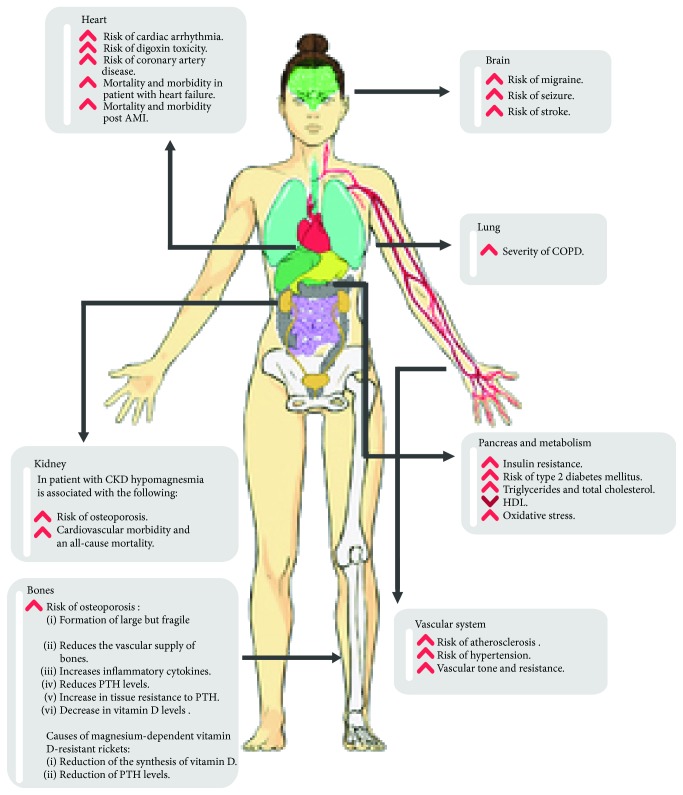
Impact of hypomagnesemia.

Symptoms of magnesium deficiency can be nonspecific and usually overlap with symptoms of other electrolyte imbalances. The severity of symptoms and signs depends on the degree of magnesium depletion and rate of magnesium decline. The symptoms usually occur when serum magnesium levels fall below 0.5 mmol/L (1.2 mg/dL) [26]. The clinical manifestations of hypomagnesemia may affect every system including neuromuscular, cardiovascular, renal, and gastrointestinal systems (Table 5) [4, 18]. The impact of chronic magnesium (Figure 2) depletion will be discussed in more depth later.

### 8.2. Causes of Hypomagnesemia

Causes of hypomagnesemia can be categorized into genetic causes [19] (Table 6) and acquired causes. The acquired causes can be attributed to decreased oral intake or GI absorption, increased renal loss, or redistribution triggered by severe illness [2]. Several medications are also known to influence serum magnesium levels by different mechanisms [4, 6, 27–31] (Table 7).

#### 8.2.1. Decreased Intake

Several dietary surveys have shown that people in North America and Europe consume less than recommended daily allowance (RDA) for magnesium as a result of food processing and the use of poor soil for agriculture [2, 5, 32]. Hypomagnesemia can also occur in times of prolonged fasting, total parenteral nutrition, or prolonged nasogastric suctioning [33].

#### 8.2.2. Impaired Gastrointestinal Absorption

Impaired gastrointestinal absorption of magnesium can be caused by a number of factors including chronic diarrhea, pancreatic insufficiency, celiac disease, chronic alcoholism, inflammatory bowel diseases, and short gut syndrome [26].

#### 8.2.3. Redistribution and Intracellular Magnesium Shift

Acute pancreatitis associated with fat necrosis can cause hypomagnesemia via saponification. Additionally, there are certain conditions that can result in an intracellular shift in magnesium distribution. These include the refeeding syndrome, pregnancy, lactation, and cardiopulmonary surgeries [26].

#### 8.2.4. Increased Renal Loss of Magnesium

Conditions such as diabetes mellitus, acute tubular necrosis, postobstructive diuresis, post kidney transplantation, excessive volume expansion, and chronic metabolic acidosis can all lead to hyperfiltration and increased renal loss of magnesium [26, 27]. Reduced renal reabsorption of magnesium can be triggered by hypokalemia, hypercalcemia, and hypophosphatemia [26]. Chronic alcoholism has been associated with reversible renal tubular dysfunction and hypomagnesemia [34]. Moreover, there are many genetic conditions (Table 6) and medications (Table 7) that have been associated with reduced renal reabsorption of magnesium.

#### 8.2.5. Drug-Induced Hypomagnesemia

Around 50 medications have been found to cause hypomagnesemia [4, 6, 27–31, 35, 36]. Table 7 lists the most commonly prescribed medications associated with hypomagnesemia.

### 8.3. Hypomagnesemia in Critically Ill Patients

Hypomagnesemia is common in critically ill patients admitted to ICU, with a prevalence between 9% and 79% in different observational studies [37–43]. It is more common in postoperative ICU patients. Hypomagnesemia in critically ill patients might be explained by many factors such as impaired magnesium absorption secondary to impaired gastrointestinal activity, malnutrition, diabetes mellitus, and other electrolyte imbalances (e.g., hypokalemia and hypocalcemia) along with medications (e.g., loop diuretics, gentamycin, and proton pump inhibitors) [41]. According to previous studies, hypomagnesemia has been shown to be strongly associated with the increased need for mechanical ventilation, increased risk of sepsis and lactic acidosis, prolonged ICU stay, and increase in mortality [43–46]. A meta-analysis including 6 studies with total of 1550 participant reported that there was a significantly higher risk of mortality (relative risk 1.9), need for mechanical ventilation (relative risk 1.56), and prolonged ICU stay in patients admitted to ICU with hypomagnesemia [47]. Several studies have shown a weak relationship between serum magnesium concentration and total body magnesium in critically ill patients. Measuring ionized magnesium was found to better represent the actual magnesium status in this group of patients [46, 48].

Due to the high prevalence of hypomagnesemia in ICU patients, it is recommended to monitor serum magnesium closely [41, 49]. Intravenous magnesium sulfate replacement has been shown to have antiarrhythmic and neuroprotective effect and might be associated with decrease mortality and length of ICU stay [50–53].

### 8.4. Hypo- and Hypermagnesemia in Hospitalized Patients

There are only a limited number of studies that have analyzed the prevalence of hypo- and hypermagnesemia in hospitalized patients in general, including non-ICU patients. A recent study from Mayo Clinic included all patients (288,120 admissions) admitted to the hospital between 2009 and 2013 and assessed the prevalence and prognostic impact of dysmagnesemia [25]. Magnesium status was evaluated in only 40% of admitted patients on the first day of admission. Of the analyzed patients, 31.5% had hypermagnesemia (Mg > 0.91 mmol/L) while 20.2% had hypomagnesemia, which was more common in patients with oncologic/hematological disorders. On the other hand, hypermagnesemia was commonly observed for patients with cardiovascular diseases, which can be attributed to the increased trend of consuming magnesium supplements in this group due to increased awareness of the beneficial impacts of magnesium on the cardiovascular system. The study found that both hypomagnesemia and hypermagnesemia were associated with increased risk of hospital mortality and prolonged length of stay when adjusted for all variables except the admission diagnosis. Since Mayo Clinic is a major referral center for the entire US continent, some of these results may not be generalizable to other hospitals.

## 9. Hypermagnesemia

Although generally rare, the prevalence of hypermagnesemia in hospitalized patients can approach >30%, and similar to hypomagnesemia, hypermagnesemia has been found to be associated with higher mortality and longer hospital stay [25]. Hypermagnesemia is usually iatrogenic and is reported along with impaired kidney function, bowel disorders, and old age. Other uncommon causes of hypermagnesemia include lithium therapy, hypothyroidism, Addison's disease, familial hypocalciuric hypercalcemia, and milk alkali syndrome [33]. Clinical consequences of hypermagnesemia vary according the serum magnesium level [54, 55] (Table 8).

## 10. Hypomagnesemia and Endocrine Diseases

### 10.1. Diabetes Mellitus

Magnesium is an essential cofactor of several enzymes involved in carbohydrate metabolism [56]. Magnesium works as an insulin sensitizer by autophosphorylation of insulin receptors and regulating tyrosine kinase activity on these receptors [57]. In addition, magnesium blocks entry of calcium into adipocytes through the L-type calcium channel. Reduced intracellular magnesium level can lead to increased calcium entry into adipocytes followed by increase oxidative stress, inflammation, and increase insulin resistance [58, 59]. On the other hand, previous studies have shown that insulin facilitates shift of magnesium from the extracellular to the intracellular space [60, 61] and reduces the tubular reabsorption of magnesium, which can lead to hypomagnesemia in people with poorly controlled diabetes and hyperinsulinemia [62].

Magnesium-deficient diet was found to be significantly associated with reduced insulin-dependent glucose uptake [63, 64] and increase incidence of diabetes mellitus [65]. Also, several studies have shown inverse relationship between serum magnesium levels and incidence of type 2 diabetes mellitus [65–67]. A meta-analysis examining the relationship between magnesium intake and type 2 diabetes involving seven cohort studies with a total of 286,668 participants concluded that four out of the seven studies showed a significant inverse relationship between magnesium intake and the risk of type 2 diabetes. It was estimated that 100 mg/day of magnesium reduces the risk of type 2 diabetes by 15% [68]. Moreover, magnesium supplements reduced the risk of developing type 2 diabetes in high-risk population, as demonstrated in a prospective study involved 2582 community-dwelling participants followed up for 7 years [69]. Oral magnesium supplementation could reduce plasma glucose levels and improve the glycemic status in a randomized controlled trial (RCT) involving 116 adults with prediabetes and hypomagnesemia [70]. Similar beneficial effects of oral magnesium have been demonstrated in people with type 2 diabetes and hypomagnesemia [71]. An observational study concluded that low serum magnesium was significantly associated with higher prevalence of diabetic nephropathy, and hypomagnesemia can be used as a marker for the risk of development of diabetic nephropathy [72]. Additionally, hypomagnesemia was associated with poor glycemic control [73], reduced HDL cholesterol, increased triglycerides, and total cholesterol levels [74, 75].

In regard to gestational diabetes, a RCT involving 70 women reported that oral magnesium supplementation provided multiple beneficial effects on metabolic status and fetal and pregnancy outcomes [76]. In addition, a recent RCT demonstrated that oral magnesium supplementation (250 mg/day) to women with gestational diabetes significantly reduced fasting plasma glucose compared with placebo. Moreover, it had beneficial effect on lipid profile by upregulating gene expression of peroxisome proliferator-activated receptor gamma (PPAR-*γ*) and glucose transporter 1 (GLUT-1) and downregulating gene expression of oxidized low-density lipoprotein receptor (LDLR) [77].

The level of magnesium was found to be lower in patients with type 1 diabetes mellitus compared to healthy individuals [78]. Hypomagnesemia was also associated with poor glycemic control [79], poor lipid profile [80], and high risk of atherosclerosis [81]. In addition, some studies in type 1 diabetes have shown a beneficial effect of magnesium supplementation in improving HbA1c and lipid profile [82] and slowing the progression of neuropathy [83].

### 10.2. Metabolic Syndrome

Several studies have linked hypomagnesemia with chronic inflammation and the metabolic syndrome. It has been proposed that hypomagnesemia can trigger low-grade chronic inflammation by contributing to activation of leukocyte and macrophage, release of acute-phase proteins and cytokines, as well as production of free radicals [59, 84, 85]. In addition, several clinical and epidemiological studies have demonstrated inverse relationships between serum magnesium and C-reactive protein (CRP) which is an important marker of inflammation [86–88]. However, it should be taken into account that oxidative stress has several causes starting with unbalanced diet involving not only magnesium but many other nutrients related with similar diseases than the ones attributed to hypomagnesemia. Nevertheless, a recent systemic review evaluated the effect of magnesium supplementation on insulin resistance. Twelve studies were identified, and it was concluded that oral magnesium supplementation had beneficial effect on insulin resistance in patients with hypomagnesemia compared to patients with normal serum magnesium [89]. Moreover, oral magnesium supplementation may have positive effect on lipid profile in individuals with and without diabetes [90, 91]. Furthermore, magnesium supplementation improves metabolic control and reduces insulin resistance in patients with type 2 diabetes and hypomagnesemia [71].

### 10.3. Magnesium and Osteoporosis

Bones store around 60% of total body magnesium, of which 30% is skeletal magnesium in the hydration shell or on the surface of hydroxyapatite [92]. Magnesium on the surface of the bones is available for exchange with serum magnesium. The remaining skeletal magnesium forms an integral part of the bones, and its release is dependent on the bone resorption [93].

Low serum magnesium has been demonstrated to be associated with low bone density in pre- and postmenopausal women [92, 94–96], and magnesium intake is found to be positively correlated with a greater bone mineral density in both men and women [97]. Furthermore, magnesium supplements have been shown to improve BMD in osteoporotic women [98, 99] and in young people [100–102]. Moreover, high dietary magnesium intake reduced prospectively the risk of osteoporotic fractures in middle-aged men and women [103]. The relationship between magnesium and bone health can be explained by different mechanisms. Low magnesium can lead to alteration of trabecular bone by formation of large but fragile crystals [104]. Moreover, low magnesium can reduce the vascular supply of bones [105] and increase inflammatory cytokines [106], which can trigger bone remodeling and osteopenia. Furthermore, reduced body magnesium can cause a reduction in PTH levels, increase in tissue resistance to PTH, and decrease in vitamin D levels [92]. On the other hand, hypermagnesemia has been associated with osteopenia and osteoporosis in postmenopausal women and people with chronic kidney diseases [92].

### 10.4. Magnesium-Dependent Vitamin D-Resistant Rickets

Magnesium acts as a cofactor for binding of vitamin D to its transport protein (vitamin D-binding protein), and it is required for conversion of vitamin D into the active form in the liver and the kidneys [2]. 1,25-Dihydroxyvitamin D has been shown to stimulate the absorption of magnesium from the intestines. Magnesium deficiency can cause magnesium-dependent vitamin D-resistant rickets by reducing the synthesis of vitamin D and impairing PTH function [2]. Adequate replacement of magnesium is essential for the treatment of magnesium-dependent rickets [107].

## 11. Hypo- and Hypermagnesemia in Cardiovascular Health

Magnesium has an important role in the regulation of cardiac rhythm, influencing vascular tone, peripheral vascular resistance, and endothelial function [3].  Table 9 summarizes the effect of magnesium on cardiovascular health [87, 108–111].

### 11.1. Risk of Arrhythmia

Hypomagnesemia is associated with an increased risk of cardiac arrhythmia via the following possible mechanisms: decreased effect of magnesium against calcium at the atrioventricular node (AV node); low magnesium causes impairment of Na^+^/K^+^-ATPase, which decreases the levels of intracellular potassium, increases intracellular sodium, and creates a less negative resting membrane potential. Both mechanisms lead to unstable membrane potentials and conduction of impulses and increase the susceptibility to arrhythmia [112]. Changes in the electrocardiograms (ECG) associated with hypomagnesemia vary according to the levels of magnesium. Mild hypomagnesemia leads to sinus tachycardia, high-peaked T waves, and ST segment depression, while severe hypomagnesemia causes shortening of the PQ interval, QRS duration, and QTc [113].

In a large scale population study, hypomagnesemia was associated with an increase in the incidence of atrial fibrillation over a 20-year follow-up [114]. Hypomagnesemia also increased the risk of atrial fibrillation postcardiac surgery [115].

Several studies have demonstrated the association between low serum magnesium and an increased risk of premature ventricular contractions, ventricular tachycardia, and polymorphic ventricular tachycardia (torsades de pointes) [112, 116–119]. A RCT showed that oral magnesium supplementation reduced the intensity of premature ventricular and supraventricular arrhythmia in patients without underlying ischemic or structural heart disease [120].

Digoxin inhibits Na^+^/K^+^-ATPase causing an increase in the intracellular concentration of sodium and calcium. Magnesium is an essential cofactor for Na^+^/K^+^-ATPase, and hence, magnesium deficiency can cause further increase in intracellular sodium, while decreasing the intracellular potassium [113]. Hypomagnesemia has been associated with increased risk of digoxin toxicity [121], which can precipitate life-threatening dysrhythmia in patients with normal digoxin and potassium level [122, 123]. It has also been shown that treatment with oral magnesium supplements may be associated with a reduction in ventricular ectopy in patients with low serum magnesium treated with digoxin for chronic atrial fibrillation [124].

### 11.2. Risk of Coronary Artery Disease

Experimental animal models have demonstrated that magnesium deficiency promotes atherosclerotic lesions in arteries, and the degree of atherosclerotic lesions was inversely related to the magnesium intake. Furthermore, low magnesium could cause endothelial dysfunction and hypercoagulability and increase the deposition of lipids and calcium in atheromatous lesions [113].

In human studies, an inverse relationship has been observed between dietary magnesium intake and serum magnesium levels and overall risk of cardiovascular diseases [125–127]. A meta-analysis on more than 77,000 cases found an inverse association between magnesium levels in the drinking water and cardiovascular mortality risk [128]. Another meta-analysis reviewing 19 studies with a total of 532,979 participants showed that dietary magnesium intake and serum magnesium concentrations were inversely associated with the risk of total cardiovascular events [111]. Moreover, hypomagnesemia was found to be associated with increased risk of coronary artery disease in a study involving 13,922 healthy subjects followed up for 4–7 years [129]. Other small RCTs have shown that oral magnesium supplementation reduced platelet-induced thrombosis [108] and improved endothelial function [130].

With regard to acute ischemia, hypomagnesemia was found to be associated with increased mortality and malignant arrhythmia in patients admitted with acute myocardial infraction. A large retrospective cohort study involving 10,806 patients with acute myocardial infraction found a U-shaped relationship between the most recent magnesium levels and mortality. Lowest mortality was seen with serum magnesium levels of ∼0.74 to 0.83 mmol/L [131]. However, several RCTs have shown conflicting results regarding the role of intravenous magnesium administration in reducing mortality in patients with acute myocardial infarction [132]. In a prospective study involving 414 patients with a median follow-up of 24 months, hypomagnesemia was associated with an increase in major adverse cardiac events in patients treated with drug-eluting stents for acute myocardial infarction [133].

Thus, there is strong evidence to support the role of magnesium in the risk of developing coronary artery diseases. Furthermore, treatment of hypomagnesemia is important in the prevention of arrhythmia in patients with acute myocardial ischemia. Previous research has suggested that consumption of water with high amount of magnesium could decrease mortality from cardiovascular disease by 30–35% [134]. Supplementing drinking water with magnesium, 25 to 50 ppm, may provide protection against cardiovascular disease and probably many other health problems [135–137].

### 11.3. Hypertension

Low dietary magnesium and hypomagnesemia might be a contributing factor in the pathophysiology of hypertension. Magnesium reduces vascular tone and resistance by enhancing vasodilator effect of nitric oxide, antagonizing the vasoconstrictor effect of calcium, bradykinin, angiotensin II, serotonin, and prostaglandin in F2*α*, and protecting the vascular endothelium through its antioxidant effect [138]. Several clinical trials have been conducted to study the effect of magnesium supplementation on the blood pressure, and at present, there is no strong evidence to support the use of magnesium supplementation in the routine management of hypertension [3, 113].

### 11.4. Magnesium and Preeclampsia

Intravenous magnesium sulfate has been used to treat preeclampsia and eclampsia for a long time. The underlying mechanisms of action can be explained by the vasodilating effect of magnesium in the vasculature and its protective role against oxidative damage during severe preeclampsia [139]. Furthermore, the anticonvulsant effect of magnesium can be explained by the role of magnesium in blocking N-methyl-D-aspartate (NMDA) receptors [3]. A recent meta-analysis concludes that magnesium sulfate for treatment of preeclampsia can reduce the risk of eclampsia by 50% [140].

### 11.5. Heart Failure

Patients with congestive heart failure are more prone to having low serum levels of potassium and magnesium due to multiple factors. These include poor oral intake, impaired gastrointestinal absorption, chronic overstimulation of the renin-angiotensin-aldosterone system, and the use of medications such as diuretics [141]. Some studies indicate the prevalence of hypomagnesemia in patients with heart failure to exceed 30% [141, 142]. A RCT has shown the association of hypomagnesemia with increased rate of ventricular ectopic beats, couplets, and episodes of nonsustained ventricular tachycardia in inpatients with heart failure. When treated for hypomagnesemia using intravenous magnesium sulfate, the same group had significantly reduced rate of arrhythmias [141]. A recent meta-analysis of seven prospective studies with total 5172 heart failure patients showed that hypermagnesemia (≥1.05 mmol/L) was associated with an increased risk of cardiovascular mortality and all-cause mortality in elderly patients with chronic heart failure and reduced left ventricular function. These findings were not observed in patients with hypomagnesemia [143].

## 12. Hypomagnesemia and Neurological Diseases

Extracellular magnesium has an inhibitory role on NMDA receptors, *γ*-aminobutyric acid (GABA) receptors, and glutamate release from NMDA receptor-rich neurons [3, 144]. Low extracellular magnesium can result in an abnormal opening of NMDA-coupled calcium channels, leading to increased calcium influx, hyperexcitability of neurons, and an increase in the production of toxic nitric oxide radicals [3, 145].

### 12.1. Headache

Observational studies have concluded that patients with migraine tend to have lower serum [146, 147] and brain [148] magnesium when compared to healthy subjects. Several mechanisms have been described to explain the relationship between low magnesium levels and migraine. Low magnesium levels can increase the aggregation of platelets and promote the secretion of serotonin, resulting in vasoconstriction which can trigger acute migraine. Furthermore, low magnesium increases neuronal excitability and triggers cortical spreading depression by increasing NMDA receptor activation, intracellular calcium, glutamate secretion, and the levels of extracellular potassium [147, 149]. Intravenous magnesium has been shown to have a beneficial additive effect in alleviating acute migraine [150, 151] and other types of acute headaches [152, 153]. To this end, oral magnesium supplements have been tried as a prophylactic agent for migraine with a significant beneficial effect [154, 155]. A recent quasi-experimental study including 70 patients concluded that both intravenous magnesium sulfate and intravenous caffeine can significantly reduce the severity of acute migraine headache, with better improvements observed with magnesium [145]. However, a meta-analysis on five RCTs with 295 patients failed to find a significant beneficial effect of magnesium sulfate in alleviating migraines, although the results need to interpreted with caution given the small sample size [156].

### 12.2. Seizures

Severe hypomagnesemia can cause a generalized tonic-clonic seizure in children and adults. Seizures are usually preceded by symptoms related to neuromuscular irritability and CNS hyperexcitability [157, 158]. Magnesium sulfate has been used as a drug of choice for seizure management, treatment, and prophylaxis in women with preeclampsia and eclampsia [157, 159–161]. It is suggested that oral magnesium supplements might have a beneficial effect when used as an adjunctive medication in the treatment of drug-resistant epilepsy [162]. The anticonvulsant effect of magnesium can be explained by its role in inhibiting NMDA glutamate receptors, increasing production of vasodilator prostaglandins, and stabilizing the neuronal membrane [157].

### 12.3. Stroke

Low magnesium intake is associated with an increased risk of stroke in several observational studies [163–165], which can be explained by the beneficial role of magnesium in endothelial function, platelet aggregation, blood pressure, and glycemic control as discussed in the previous section. Patients suffering acute ischemic stroke and admitted with low magnesium levels have an increased inpatient mortality risk [166] and increased intensity of neurological deficit [167]. This might be attributed to the cerebral vasoconstriction triggered by hypomagnesemia. On the other hand, a large double-blinded RCT reported that intravenous magnesium sulfate administration for patients with acute stroke within 2 hours of onset of stroke symptoms had no impact on the improvement of disability outcomes at 90 days of poststroke [168].

## 13. Hypomagnesemia and Respiratory Diseases

Several studies have indicated that dietary magnesium and intravenous magnesium sulfate infusion were associated with an improvement in lung function, as measured by forced vital capacity (FVC) and forced expiratory volume (FEV) [169, 170]. While the mechanism of action is not entirely understood, it is possible that magnesium acts via anti-inflammatory effect and reduces lung inflammation along with the role of magnesium in regulating bronchoconstrictors such as acetylcholine (ACh) and histamine, as well as the vasodilatory and the bronchodilatory effect of magnesium [3].

### 13.1. Bronchial Asthma

A single dose of intravenous magnesium sulfate (1.2 g) has been recommended for the management of acute severe and life-threatening exacerbation of asthma [171]. A Cochrane review analyzing the findings of 14 RCTs including 2313 adult patients presented to the Emergency Department with acute exacerbation of bronchial asthma concluded that intravenous magnesium sulfate reduced the need for hospital admission and improves lung function test [172]. Another RCT also showed that the use of inhaled isotonic magnesium as adjuvant therapy in treatment of severe acute exacerbation of bronchial asthma was associated significant improvement of FEV1 at 90 minutes [173]. The role of magnesium in antagonizing the effect of calcium and altering intracellular cAMP and thereby reducing the neutrophil respiratory burst is believed to be the mechanism of action of magnesium sulfate, which helps in controlling airway inflammation during asthma [174].

### 13.2. Chronic Obstructive Pulmonary Disease (COPD)

Hypomagnesemia is associated with advanced chronic lung diseases, increased severity of the disease, and the length of hospital stay, according to observational studies [175, 176]. However, a systemic review of four RCTs has failed to show any significant therapeutic effect of intravenous or inhaled magnesium used in the treatment of COPD [177]. Overall, there are only a few experimental trials evaluating the effect of magnesium on COPD, and the existing trials have several limitations [178]. Therefore, further studies are required to elaborate the impact of magnesium on COPD.

## 14. Magnesium Disorders and Kidney Disease

The kidney has a very important role in magnesium homeostasis. Mild and moderate renal impairment can increase the fractional excretion of magnesium to compensate for the loss of glomerular filtration [179]. However, with advanced chronic kidney disease (creatinine clearance < 30 mL/min), this compensatory mechanism fails to maintain the homeostasis, resulting in hypermagnesemia [180]. In addition, magnesium homeostasis can be affected by vitamin D, PTH, and calcium abnormalities associated with advanced chronic kidney disease. In peritoneal hemodialysis patients, the use of higher concentration of magnesium dialysate can cause hypermagnesemia [179, 180]. In contrast, hypomagnesemia might be observed in patients as a result of the use of low magnesium dialysate, secondary to medications used or as a result of an underlying medical problem such as malnutrition or alcohol abuse [181]. Patients with chronic kidney disease and hypomagnesemia may have higher risk of osteoporosis [182], cardiovascular morbidity, and an all-cause mortality [183].

## 15. Future Perspectives

Further research would be valuable in assessing the utility of magnesium level as a marker of disease severity, especially in hospitalized patients. More importantly, much previous research focused on diseases caused by hypomagnesemia and the therapeutic role of magnesium. However, many diseases discussed in this review could be a reflection of the modern magnesium-deficient diet. We suggest that more focus should be on the preventive role of magnesium on alleviating the burden of disease.

## 16. Conclusion

Magnesium is an essential cation involved in numerous enzymatic reactions and important for many vital physiological functions. Magnesium disorders, especially hypomagnesemia, are common in clinical settings and are associated with many adverse health outcomes. Magnesium has been used successfully in treatment of medical conditions such as bronchial asthma, cardiac arrhythmia, eclampsia, and preeclampsia, and oral magnesium supplements have indicated beneficial health outcomes. Further research is needed to evaluate to feasibility and effectiveness of magnesium supplementation on overall morbidity and mortality.

## Figures and Tables

**Table 1:** Role of vitamins and hormones in magnesium homeostasis.

Hormone/vitamin	Role of hormones/vitamins	Comments
Vitamin D	1,25-Dihydroxyvitamin D3 can stimulate intestinal magnesium absorption.	Mg is required for metabolism of vitamin D in the liver and the kidneys and also for its transportation in serum.
PTH	Helps in the reabsorption of Mg in the kidney, absorption in the gut, and release from the bone.	Hypercalcemia interferes with the role of PTH in magnesium regulation.
Estrogen	Enhances Mg reabsorption in the kidney and absorption in the gut by stimulating TRPM6 expression.	

Mg: magnesium; PTH: parathyroid hormone.

**Table 2:** Role of magnesium in the human body.

*Cofactor for enzymes involved in*
Protein synthesis, muscle and nerve transmission, neuromuscular conduction, and blood glucose and blood pressure regulation.
*Role in active transport*
Facilitates active transport of calcium and potassium ions across cell membranes, which is essential for the conduction of nerve impulses, muscle contraction, maintaining vasomotor tone, and normal heart rhythm.
*Structural roles*
Important for the structure of bones, proteins, many enzymes, mitochondria, DNA, and RNA.
*Role in immunological functions*
Involved in macrophage activation, adherence, and bactericidal activity of granulocyte oxidative burst, lymphocyte proliferation, and endotoxin binding to monocytes.

**Table 3:** Assessment of magnesium status.

Test	Comments
Serum magnesium	Sometimes not adequate since less than 0.3% of total body magnesium is found in serum. However, it is easy, accessible, and cheap.

24 hours excretion in urine or the fractional excretion of magnesium	Helps in differentiating renal wasting of magnesium from inadequate intake or poor absorption as an etiology for hypomagnesemia.

Magnesium loading test	Identifies patients with normomagnesic magnesium deficiency.
	Assesses intestinal absorption of magnesium.
	Indirectly assesses bone status of magnesium.

Magnesium concentration in RBCs	Can give early indication of magnesium deficiency.

Isotopic analysis of magnesium	Assesses the absorption of magnesium from the gastrointestinal tract in research setting.

Ionized magnesium	More accurate, especially in critically ill patients with rapid change in hemodynamics.
	Not effected by low albumin.

RBCs: red blood cell counts.

**Table 4:** Prevalence of hypomagnesemia in different populations and under different clinical settings.

Authors	Country and year	Definition	Sample	Sample size	Prevalence
Chernow [23]	USA, 1989	<0.75 mmol/L	ICU	193	61%
Schimatschek and Rempis [24]	Germany, 2001	<0.76 mmol/L	Unselected population	16,000	14.5%
Cheungpasitporn et al. [25]	USA, 2015	<0.70 mmol/L	Hospitalized patients	65,974	20.2%

ICU: intensive care unit.

**Table 5:** Clinical and laboratory manifestations of hypomagnesemia.

System	Manifestations
Neuromuscular	Tremors, muscle fasciculation, muscle spasms and cramps, muscle contractions, numbness, tingling, and weakness.
Central nervous	Agitation, depression, sudden change in behavior, encephalopathy, and seizures.
Cardiovascular	Cardiac arrhythmia and ECG changes.
Gastrointestinal	Loss of appetite, nausea, and vomiting.
Metabolic	Hypokalemia and hypocalcemia.

**Table 6:** The most common genetic disorders causing hypomagnesemia.

Disorder	Inheritance	Gene	Other features (other than hypomagnesemia)
*Hypercalciuric hypomagnesemias*			Hypercalciuria and nephrocalcinosis
FHHNC type 1	AR	CLDN16	Polyuria/polydipsia, elevated serum PTH, and renal failure
FHHNC type 2	AR	CLDN19	Besides FHHNC type 1 features, patient has ocular abnormalities
ADHH Bartter syndrome type 5	AD	CASR	Hypocalcemia with normal or low PTH
Bartter syndrome type 3 (classical type)	AR	CLCNKB	Gitelman-like phenotype possible, rarely nephrocalcinosis

*Gitelman-like hypomagnesemias*	Hypocalciuria, hypokalemia, and metabolic alkalosis		
Gitelman syndrome	AR	SLC12A3	Chondrocalcinosis at older age
ADTKD/RCAD	AD	HNF1B	Renal, genital, and pancreatic abnormalities

*Mitochondrial hypomagnesemias*			
KSS	Mt	Mitochondrial deletion	External ophthalmoplegia, retinopathy, and cardiac conduction defects

ADHH: autosomal dominant hypocalcemia with hypocalciuria; ADTKD: autosomal dominant tubulointerstitial kidney disease; FHHNC: familial hypomagnesemia with hypocalcemia and nephrocalcinosis; RCAD: renal cysts and diabetes; KSS: Kearns-Sayre syndrome; AR: autosomal recessive; AD: autosomal dominant.

**Table 7:** Medications associated with hypomagnesemia.

Medications	System	Pathophysiology
Aminoglycoside antibiotics	Renal	Impair renal tubular reabsorption ± acute tubular necrosis (ATN)
Amphotericin B	Renal	Renal toxicity and impaired magnesium reabsorption
Antiepidermal growth factor (EGF) receptor (e.g., cetuximab)	Renal	Impairs magnesium reabsorption
Calcineurin inhibitors (e.g., cyclosporine and tacrolimus)	Renal	Impair magnesium reabsorption
Platinum derivatives (e.g., cisplatin and carboplatin)	Renal	Impair magnesium reabsorption
Loop and thiazide diuretics	Renal	Impair magnesium reabsorption
Pentamidine	Renal	Impairs magnesium reabsorption
Proton pump inhibitors (PPI)	GI	Reduce intestinal absorption of magnesium by downregulating the TRPM6 transporters.

GI: gastrointestinal.

**Table 8:** Clinical manifestations of hypermagnesemia.

Serum magnesium levels	Manifestations
0.70–1.0 mmol/L	Normal level.
2.2–3.5 mmol/L	Nausea, vomiting, facial flushing, urinary retention, ileus, and hypotension.
3.9–5.2 mmol/L	Somnolence, absence of the deep tendon reflex, and complete heart blockage.
>6.5 mmol/L	Respiratory depression, paralysis, and complete heart blockage.
>8.7 mmol/L	Asystole.

**Table 9:** Magnesium effects on the cardiovascular system.

Improvement in endothelial function.
Induction of direct and indirect vasodilation.
Improvement in blood pressure.
Beneficial effects on arrhythmias, inflammatory reactions, and platelet aggregation.
Potential effect in improving exercise tolerance in patients with stable coronary artery disease.
Improvement of insulin homeostasis and lipid metabolism.
Reduces platelets activation and thrombosis.
Reduces cellular ischemic injury by reducing calcium overload in coronary arteries.
